# Bioactive Potential of Aqueous Phenolic Extracts of Spices for Their Use in the Food Industry—A Systematic Review

**DOI:** 10.3390/foods12163031

**Published:** 2023-08-12

**Authors:** Carmen Duque-Soto, Ana Ruiz-Vargas, Ascensión Rueda-Robles, Rosa Quirantes-Piné, Isabel Borrás-Linares, Jesús Lozano-Sánchez

**Affiliations:** 1 Department of Food Science and Nutrition, University of Granada, Campus Universitario s/n, 18071 Granada, Spain; carmenduque@ugr.es (C.D.-S.); aruivar2@correo.ugr.es (A.R.-V.); ruedarobles@ugr.es (A.R.-R.); jesusls@ugr.es (J.L.-S.); 2 Research and Development Functional Food Centre (CIDAF), Health Science Technological Park, Avenida del Conocimiento 37, Edificio BioRegión, 18016 Granada, Spain; rquirantes@cidaf.es; 3 Department of Analytical Chemistry, Faculty of Sciences, University of Granada, 18071 Granada, Spain

**Keywords:** phenolic compounds, plant extracts, medicinal and aromatic plants, antioxidant activity, antimicrobial activity, green extraction

## Abstract

The interest on the use of natural sources in the food industry has promoted the study of plants’ phenolic compounds as potential additives. However, the literature has been focusing on essential oils, with very few studies published regarding aqueous extracts, their phenolic composition, and bioactivity. A systematic review was conducted on different databases following PRISMA guidelines to evaluate the relevance of the phenolic content of different aromatic spices (oregano, rosemary, thyme, ginger, clove, and pepper), as related to their bioactivity and potential application as food additives. Although different extraction methods have been applied in the literature, the use of green approaches using ethanol and deep eutectic solvents has increased, leading to the development of products more apt for human consumption. The studied plants present an interesting phenolic profile, ranging from phenolic acids to flavonoids, establishing a correlation between their phenolic content and bioactivity. In this sense, results have proven to be very promising, presenting those extracts as having similar if not higher bioactivity than synthetic additives already in use, with associated health concerns. Nevertheless, the study of spices’ phenolic extracts is somehow limited to *in vitro* studies. Therefore, research in food matrices is needed for more understanding of factors interfering with their preservation activity.

## 1. Introduction

The production of millions of tons of residues every year as a result of a modern lifestyle and its impact on the environment has become a rising public worry. In response, society has focused on the development of more sustainable and healthy approaches. Thus, the use of natural agro-food by-products as sources in the industry has been promoted as a great alternative to currently used raw materials [1]. This has also transpired into the food industry, where consumers seem to increasingly prefer more natural additives as opposed to their synthetic counterparts, influenced by the recent concerns about their health safety.

Medicinal and aromatic plants (MAPs) have been historically used for traditional medicine and pose as an interesting source of bioactive compounds, with proven antioxidant and antimicrobial activities [2]. Therefore, they can potentially be used in the food industry for the delay or prevention of food quality loss caused by microbial growth and undesirable chemical reactions [3].

Some of the most renowned MAPs are spices, many of which have been recognized as safe for human consumption, such as oregano, parsley, rosemary, or thyme. They are mainly used in food preparations as flavourings and as alternatives for salt consumption for people with high blood pressure [4]. Additionally, their consumption has been related to protection against cardiovascular diseases, neurodegeneration, type II diabetes, and cancer [5].

Spices have already been used as raw materials for a variety of industries (including cosmetic, pharmacological, and food industries) as flavourings, food colourings, essential oils, sweeteners, or even for their nutraceutical activities, mainly due to their phenolic composition [6,7,8]. Along with the increasing interest for the consumption of more natural and environmentally friendly foods, spices are gaining a newfound interest for their innovative use as a source for these natural bioactive compounds [9].

Phenolic compounds are plant secondary metabolites consisting of an aromatic ring with one or more hydroxyl groups and can be synthesized using the shikimic acid or acetate pathway [10]. More than 8000 phenolic compounds have been described in MAPs, which are commonly classified as phenolic acids, flavonoids, stilbenes, and lignans [11]. In this regard, the most popular characterization technique has been high-performance liquid chromatography (HPLC), although the Folin–Ciocalteu method has also been used to determine total phenolic compounds.

It must be considered that bioactive compounds can be lipophilic, such as essential oils, oleoresins, curcuminoids, and carotenoids that are extracted using non-polar solvents, or hydrophilic, such as many polyphenols that are extracted using more polar solvents [12]. As well-known important sources of phenolic compounds and profoundly reviewed in the previous literature, essential oils have been widely studied as additives for food preservation [13,14]. However, the inherent characteristics of essential oils constrain their composition primarily to the least polar polyphenols, which are also present in relatively low concentrations. This circumstance could potentially limit their practical utility. In contrast, the use of aqueous-based extracts rich in more polar phenolic compounds, which appear in higher concentrations, is, therefore, very promising.

Phenolic compounds are known for their antioxidant and antimicrobial activity, which have sustained their interest in the food industry [15,16,17]. The bioactivity of phenols is an interesting topic for their use as food preservatives. Its potential mainly depends on their content and distribution in the plant, which is affected by factors prevailing during the cultivation of plants, such as the weather, type of soil, type of crop, and sun exposure, among others [18]. The addition of these compounds to the packaging for antimicrobial protection has already been studied in various forms: (a) in special structures containing them, (b) either in their volatile or non-volatile form, as polymers, (c) as polymer coating, (d) bound through ionic or covalent bonds, or (e) intrinsically in the packaging. In a similar way, for their control of lipid and pigment oxidation, they have been studied in foods as (a) an edible coating or (b) freed on its surface [19]. With the intention of extracting and concentrating these compounds, implementation of both conventional and more advanced extraction techniques have been studied [18].

As far as we know, this is the first review regarding the comparison of the extraction techniques available and the evaluation of the most recent methods, focused on a more environmentally friendly and safer approach to human consumption, as well as a study of their phenolic content as related to their bioactivity and potential use, as essential aspects into their legal consideration as additives [20]. As previously started, the study of phenolic compounds from spices has been focused on their essential oils. For this reason, even though they present great potential as sources of more polar and abundant compounds, aqueous phenolic extracts have not been considered until recently, as their phenolic characterization is rather scarce. Also, there is a dire need to evaluate the effect that different extraction methods and conditions could have on the chemical composition of extracts and its relevance to its potential synergistic bioactivity. Additionally, previous evidence of their bioactivity and safety for application in food products is needed in order to enable their legal use in the industry, as stated by current legislation and mediated by official organizations, such as the European Food Safety Authority (EFSA) and the Food and Drug Administration (FDA) [20].

The aim of this systematic review is to evaluate the evidence regarding the preservative activity of phenolic compounds from MAPs extracts, such as oregano (*Origanum vulgare, Origanum glandulosum*), rosemary (*Rosmarinus officinalis* L.), thyme (*Thymus zygis gracilis, Thymus membranaceus, Thymus longiflorus*), ginger (*Zingiber officinale*), clove (*Eugenia caryophyllata*), and pepper (*Piper nigrum*), which may promote their use in the food industry. This main objective can be divided into the following sub-objectives: (1) determining phenolic compounds content in plant extracts; (2) analyzing antioxidant activity of polyphenol-rich extracts; and (3) studying antimicrobial activity of those extracts.

## 2. Materials and Methods

### 2.1. Search Strategy

Systematic research was conducted following PRISMA guidelines. PubMed and Scopus databases were searched to select papers, including the phenolic composition of extracts and their bioactivity in *in vitro* and food-applied studies. This review focuses on systematic reviews, meta-analyses, and randomized controlled studies published in the years 2001–2022. No language filter was applied. Exclusion criteria were: (1) restricted access articles, (2) non-relation to the subject of study, and (3) articles focused on spices’ essential oils.

In addition, the PICO process was followed using the following parameters: (1) Population: *in vitro* analysis; (2) Intervention: antioxidant and antimicrobial activity; (3) Comparison: human food; and (4) Outcome: aromatic plants’ use with the technological function being possible, sustainable, and innovative.

**Figure 1:**
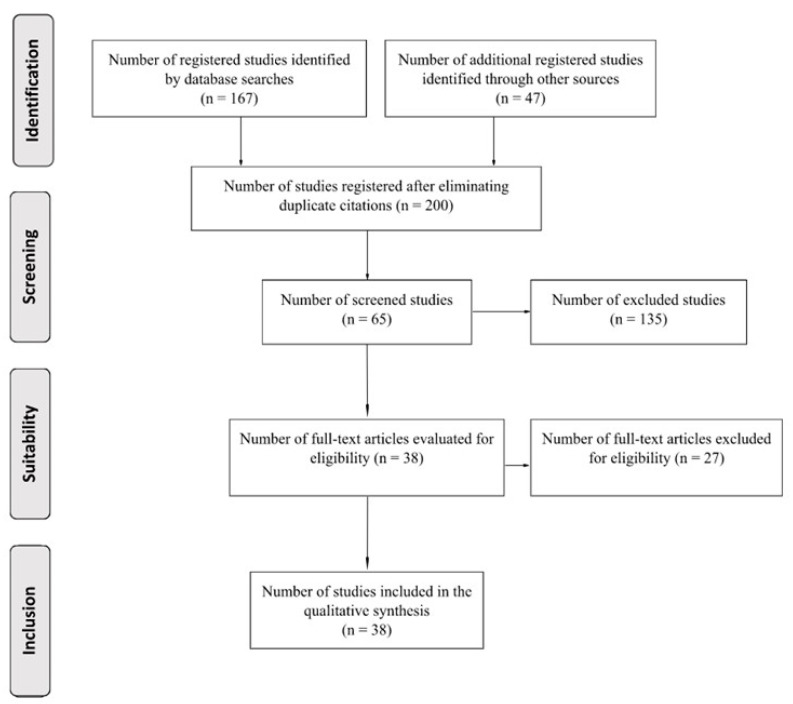
PRISMA scheme with articles included in the present systematic review.

As for eligibility criteria, articles not focused on the subject of interest were excluded from the analysis, including articles evaluating essential oils and those that did not include the selected plant families. The study selection process is shown in Figure 1.

### 2.2. Search Formulas and Keywords

The search formulas and keywords used in the present systematic review have varied depending on the interest at hand: (a) For phenolic compounds: (Herbs OR Spices OR Aromatic plants OR plant extract) AND (Phenol compounds OR polyphenols OR bioactive compounds) AND (Extraction methods OR extraction OR characterization methods) AND (green extraction OR alternative technologies); (b) For antioxidant activity: (Herbs OR Spices OR Aromatic plants OR plant extract) AND (Phenol compounds OR polyphenols OR bioactive compounds) AND (Antioxidants OR Natural antioxidants) AND (Oregano OR *Origanum* OR Thyme OR *Thymus serpyllum* OR *Thymus* plant OR Rosemary OR Ginger OR Clove OR Pepper); (c) For antimicrobial activity: (Herbs OR Spices OR plant extract) AND (Phenol compounds OR polyphenols OR bioactive compounds) AND (Antimicrobial OR Antiviral) AND (Food-preservation OR Food safety).

## 3. Results and Discussion

### 3.1. Main Phenolic Compounds Identified in Medicinal and Aromatic Plants

In this review, the most prominent phenolic characterization studies and extraction techniques have been compiled. The selection of a specific method and extraction technique has depended on the physico-chemical nature of the compounds to be extracted, its cost, and security. The factors that influence the extraction efficiency are the chemical nature of the extraction solvent, the particle size of the plant materials, the solvent–solid ratio, temperature, and extraction time. Additionally, extract storage conditions have influenced its shelf life and bioactivity [2].

Extraction techniques applied in the literature have been (a) conventional techniques; (b) pressurized liquid extraction, as supercritical fluids (PLE), subcritical fluids (SFE), and accelerated solvents; (c) microwave-assisted extraction (MAE); or (d) ultrasound-assisted extraction (UAE). However, microwave- and ultrasound-assisted extraction have been the most widely used techniques for bioactive compounds. The latter allows the extraction of stronger flavors and their preservation for a longer time [21]. In addition, its non-thermal nature allows a lower negative effect on basic physical and chemical parameters, such as total acid, total soluble solid, color, electrical conductivity, viscosity, and density [22].

Although a multitude of solvents from glycerol to choline chloride have been applied, the highest performing and, therefore, most used are ethanol and methanol. This has been observed in Bellumori et al. (2016), where the use of ethanol and acetone as extraction solvents in ultrasound-assisted extraction (UAE) and microwave-assisted extraction (MAE) improved the extraction yield of phenolic compounds, obtaining better results than when a double ethanol maceration as solid–liquid extraction processes was applied [23].

The importance of the selected solvent can be further observed in Proestos and Komaitis (2006), where ultrasonically assisted extraction using water as a solvent reduced the amounts of phenolic compounds as compared to the conventional method [24]. Different extraction solvents were compared: 60% methanol, 60% acetone, water, and ethyl acetate/water (60:30, *v*/*v*), with methanol being the best option for extracting phenolic compounds from plant tissues. These results were in line with those previously published by Wang, Provan, and Helliwell (2004) [25]. In this study, ethanol, methanol, acetone, and acetonitrile, all at 30% in water (*v*/*v*), were evaluated for the extraction of rosmarinic and caffeic acids and compared with the extraction using water as the selected solvent. Extraction was similar when using ethanol, methanol, acetonitrile, or acetone. However, rosmarinic acid content was 20% lower when compared with the extraction using water as a solvent. This has also been observed in Bellumori et al. (2016), where a low extraction of total phenolic content (TPC) was reported when water was used as a solvent [23]. Additionally, the efficiency of methanol and ethanol as extraction solvents was confirmed in Švarc-Gajić et al. (2013), where 70% methanol provided the highest yield of TPC with MAE, while flavonoids were extracted more efficiently in the microwave field using 70% ethanol [26]. However, an assessment of different extraction solvents has been restricted to specific studies and, while it would be ideal to evaluate this effect through the literature, differences in the experimental conditions applied for each extraction technique and the evaluated spice between studies do not allow for this comparison to be carried out.

Also, the use of a combination of emerging technologies has presented a great potential for higher extraction than using these individual techniques [27]. This has been observed in Tzima et al. (2021) where Pulsed Electric Field was used as a pre-treatment step for improving the obtention of phenolic compounds using UAE [28]. This effect was explained through an increase in solubility of phenolic compounds and facilitated permeability through plant cell walls, facilitating enhanced recovery of ultrasound-extracted bioactive compounds.

Even though the aforementioned methods provide good extraction results, their adequacy for the implementation in food products must be addressed. In this sense, the development of the so-called green methods has taken interest, not only as more environmentally friendly approaches but also as more suitable for their later purpose. Regarding green methods for the extraction of bioactive compounds, one of the most popular is the use of deep eutectic solvents (DES). In Barbieri et al. (2020), combinations of glycerol:choline chloride (1:2 *v*/*w*) and water (10% *w*/*w*), lactic acid:choline chloride (1:3 *v*/*w*) and water (10% *w*/*w*), 1,2-propanediol:choline chloride (1:2 *v*/*w*) and water (10% *w*/*w*), and oxalic acid:choline chloride (1:1 *v*/*w*) and water (10% *w*/*w*) were used and compared with ethanol as a solvent [29]. In this case, the combination of solvents showed a higher antioxidant capacity than the alcoholic extract, measured with the 2,2-diphenyl-1-picrilhydrazil oxide depletion (DPPH) and ferric-reducing antioxidant capacity (FRAP) assays. In addition, ethanolic extract presented the lowest stabilizing ability of the phenolic compounds present in the extract. Overall, these promising results propose the use of GRAS (generally recognized as safe by the FDA) solvents as great alternatives for the obtention of enhanced extracts in the food industry.

Regarding the plant material, phenolic compounds can be extracted from multiple parts of the aromatic plant, such as flowers, leaves, or seeds. However, as phenolic content seems to vary between different plant structures, flower extracts seem to generally present the highest presence of polyphenols [30]. However, other structures have also been used. In the analyzed studies, rosemary polyphenols were extracted from leaves [29,31] and branches [32]. Oregano phenolic extracts were mainly obtained from leaves [32]. As for thyme, extracts were obtained either from the leaves [31,33,34] or the leaves and branches [32]. Rosemary extracts were obtained from the rhizome area [35,36] and, in the case of cloves, from flowers [33]. The rest of the studies do not indicate the part of the aromatic plant from which the extracts were obtained.

As for characterization methods, the most used among the literature were HPLC and the Folin–Ciocalteu method [23,26,30,33,36,37,38,39,40,41,42,43,44,45,46,47,48]. The combination of the former coupled with the mass spectrometry has also been widely used as an analytical characterization method. Among HPLC, normal phase (NP-HPLC), reverse phase (RP-HPLC), and hydrophilic interaction liquid chromatography (HILIC-HPLC) have also been used [49].

Throughout different studies, regardless of the extraction method, the phenolic profile of spices seems to be comparable. Main phenolic compounds characterized in different spices can be found in Table 1.

**Figure 2:**
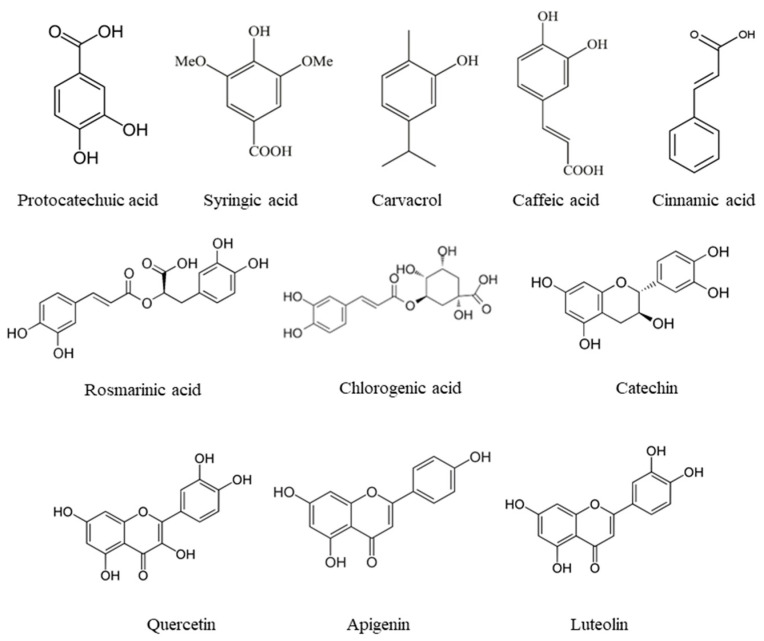
Most relevant phenolic compounds in different spices.

The main phenolic compounds identified in the different species of oregano were similar, although variations in their content may be present. Some of the main phenolic compounds identified in this spice belonged to phenolic acids, such as rosmarinic acid, caffeic acid, gallic acid, *p*-coumaric acid, chlorogenic acid, and ferulic acid [24,36,38,43,48]. Structures of the most relevant phenolic compounds found in the spices are presented in Figure 2. The mentioned acids are widely known for their great antioxidant activity. The presence of flavonoids, such as apigenin, luteolin and quercetin, should also be noted [24,38,48].

Rosemary extracts were the most studied in terms of optimization of the extraction process and characterization of bioactive components, which could be related to their industrial use. This is also observed for thyme and oregano, widely studied in comparison to other spices, such as cloves and ginger, from which no significant results have been found. The main phenolic compounds identified in rosemary were carnosoic acid and rosmarinic acid. Extracts of this spice contain flavonoids, like catechin, quercetin, rutin, naringin, and kaempferol, and phenolic acids, like chlorogenic acid, p-hidroxibenzoic acid, and protocatechuic acid [23,32,33,42].

Thyme extracts presented rosmarinic acid, caffeic acid, cinnamic acid, chlorogenic acid, and ferulic acid, among other compounds [34,37,38,43]. The main flavonoids contained were apigenin, luteonin, and quercetin. Phenolic content has been abundantly quantified using the Folin–Ciocalteu method, but there is not much information about the quantification of individual phenolic compounds, as well as a lack in general characterization of this genus [38].

As for pepper, its main phenolic compounds were capsaicinoids, such as nordihydrocapsaicin, capsaicin, dihydrocapsaicin, homocapsaicin, and homodihydrocapsaicin [44,50]. These bioactive compounds were extracted using ultrasound using methanol as the extractant solvent and characterized using HPLC with fluorescence detection and monolithic columns for separation [44].

It is important to note that since studies evaluating different solvents found no differences between ethanol and methanol, ethanol should be preferably selected for food applications due to its GRAS solvent status. For its application in the industry, suitability of these extracts for human consumption needs to be considered. All substances isolated in the considered extracts and most solvents used for their extraction in the considered studies are GRAS substances and, thus, initially considered as innocuous, making them a good alternative to chemical additives normally used in the food industry.

### 3.2. Antioxidant Capacity Analysis in Spices

#### 3.2.1. Main Antioxidant Methods Applied to Aromatic Plants

An abundance of antioxidant activity assays has been applied on spices in the literature. These methods can be based on hydrogen atom transfer, such as Oxygen Radical Absorbance Capacity (ORAC) or Total Radical-Trapping Antioxidant Parameter (TRAP); electron transfer, such as FRAP; or in a mix of both phenomena, such as depletion of 2,2’-Azinobis-3-ethyl-benzothiazoline-6-sulfonic acid (ABTS) or DPPH. In order to measure the antioxidant activity of extracts accurately, choosing an adequate assay based on the chemicals of interest and, above all, the food matrix in which it will be included are critical.

ABTS and DPPH assays seem to be the most used radical scavenging assays for spices [30,36,38,41,43,51,52]. This can be related to the convenience and adequacy of these methods to a variety of conditions, as well as their benefits. DPPH stands out as a fast, simple, and inexpensive assay, with few steps and reagents needed, as well as with a high repeatability. Some disadvantages may be the use of organic solvents, their sensitivity to pH, and color interference that could cause underestimation of results [53].

On the other hand, ABTS can be applied to either lipophilic or hydrophilic samples, with high reproducibility, which makes it a highly versatile assay. This method may also have some disadvantages, such as its sensitivity to light and temperature or lack of standardization, which makes it difficult to compare in interlaboratory studies [53].

Even though relatively less is used, ORAC, TRAP, and FRAP have also been described in the literature. As for ORAC, its relevance for the study of biologically relevant free radicals as well as the use of various free radical generators can be interesting, but results cannot be expressed in weight, hindering interstudy comparisons. On another note, TRAP may be suitable for studying activity against potent antioxidants (such as hydroxyl and peroxyl radicals). Lastly, FRAP must also be considered as an easy and inexpensive assay, although instability of oxygen electrodes, the use of low pH, or the lack of detection of SH-group antioxidants may cause some disadvantages [53,54,55].

However, a combination of a few assays must be carried out to have a realistic assessment of the antioxidant capacity of a sample. Moreover, the difference in nature between some of the assays as well as the expression of the results based on different standards can hinder the comparison of different results and studies.

#### 3.2.2. Antioxidant Activity and Main Phenolic Compounds Identified in Aromatic Plants

As has been established in the literature, the antioxidant activity of medicinal and aromatic plants shows great potential for their use in the food industry, which is evidence for their acceptance as food additives. In this sense, antioxidant potential of medicinal and aromatic plants has been studied through their phenolic composition, as main contributors to this activity, as presented in Table 1 and Table 2. Phenolic content of extracts of multiple MAPs obtained through different extraction procedures has been evaluated, as is the case for *Lamiaceae* plants, such as oregano, rosemary, and thyme. Additionally, several studies have considered their application on a variety of food matrices.

Antioxidant potential of oregano has been evaluated in extracts and applied on different food matrices through ABTS and DPPH methods [36,38,43]. As for extraction conditions for phenolic recovery, MAE appears to be one of the main techniques. In Nabet et al. (2019), MAE phenolic extracts from *O. glandulosum* showed good antioxidant potential [38]. When phenolic compounds were extracted with MAE, lower concentrations of ethanol resulted in greater recovery of compounds and enhanced antioxidant activity. Studies on other species of oregano (*O. vulgare*) presented similar consistent high-antioxidant results [36,43]. Nevertheless, the main phenolic composition appears to be similar throughout the studies, though total content may vary. Many of the identified compounds belong to phenolic acids, such as caffeic or rosmarinic (known for its great antioxidant capacity) as well as flavonoids, such as quercetin, apigenin, and kaempherol, mainly in its glycosylated forms.

Additionally, oregano extracts have been successfully applied in food matrices. In Table 3, studies of different spices included in food matrices with antioxidant activity are presented, as well as a comparison with other common antioxidant additives. In this sense, the use of polyphenolic-rich extracts provided a greater antioxidant effect in cooked meat than in raw meat, and meat proteins greatly affected antioxidant activity [59].

Rosemary extracts exhibit consistent high-antioxidant activity through different assays and studies [30,36,41,43]. However, the best antioxidant results were obtained for ABTS and FRAP in Teruel, Garrido, Espinosa, and Linares (2015) [41]. This may be influenced by the extraction method and conditions applied, which differed for all studies. The antioxidant potential of the extracts was further studied in Costa et al. (2015), in which a comparison of the antioxidant activity of extracts obtained through SFE using volatile oil and oleoresin showed in both cases better activity than other natural antioxidants used as a control (betacarotenes and linolenic acid) [64]. In these studies, the phenolic profile seems to be similar, presenting phenolic acids (such as caffeic acid and derivatives), carnosic acid, carnosol, and flavonoids.

These results have proven the potential of these extracts and have, therefore, established the interest of the study of the effectiveness of rosemary extracts on the oxidative protection of food matrices. In fact, different studies have considered their application on meat matrices, as can be observed in Table 3. Their phenolic extracts have been previously evaluated by the EFSA and approved by the European Union for their use as a dietary antioxidant additive [20]. These extracts have been mainly evaluated in different meat-based foods, with an effective protection against lipid and protein oxidation under storage conditions [41,47,60,61,65]. Moreover, rosemary extracts have proven to be more efficient than synthetic antioxidants butylhydroxyanisole (BHA) and butylhydroxytoluene (BHT) for the prevention of higher substances reactive to the thiobarbituric acid (TBARS) value or color loss in frozen raw sausages, which will be further discussed below [61]. In Karre et al. (2013), rosemary phenolic extracts protected efficiently turkey meat, vacuum-packaged ground beef and pork meat, and ground cooked beef from oxidation [66].

Similarly, free radical scavenging of thyme extracts seems to be similar between different studies and species considered [36,43,52,67]. The best antioxidant results for this MAP were presented in Nabet et al. (2019) for ABTS, when lower ethanol concentrations were applied in MAE [38]. Antioxidant activity of thyme extracts has been compared in the literature to additives already in use. In this sense, in Roby, Sarhan, Selim, and Khalel, (2013), thyme methanolic extracts showed higher antioxidant activity than additives, such as BHA, tert-butylhydroquinone (TBHQ), and α-tocopherol [34]. In Mascoloti et al. 2022, thyme extracts presented promising antioxidant activity, with the ability to counteract lipid peroxidation in TBARS assays with an IC50 under 26 ug/mL [68].

The literature is sparse regarding the study of antioxidant activity of ginger extracts. In this sense, antioxidant activity of different extracts was evaluated in different formulations, and the obtained results from the highest to the lowest activity are the following: dry ginger > grilled ginger > charred ginger > fresh ginger [69].

As for clove, its phenolic content seems to vary in comparison to other *Lamiaceae* spices. These extracts contained volatile compounds, including eugenol and tannin, not found in other analyzed extracts [36]. Clove extracts have also been studied in food matrices, focusing on their use in different meat products, where they have proved to have a high-antioxidant effect by reducing protein and lipid oxidation, as compared with common additives, and will be further discussed in following sections [47,62]. Although fresh options seem to be highly accepted by consumers, dry clove extracts have exhibited a more promising bioactivity [30].

The reviewed literature shows the great variation and difficulty in the comparison of the antioxidant activity results present in the literature and its relation to the plant’s phenolic content. Additionally, variations in different spices and studies may be caused by multiple factors, such as differences in cultivars, seasons, or sun exposure, that may result in an altered phenolic profile [70]. The use of different assays and extraction conditions as well as the lack of a standardization only emphasizes that predicament.

Throughout all the spices studied, there seems to be a consistency among phenolic compounds identified. The main compounds found in these MAPs belong to phenolic acids, as rosemary and caffeic acids have been found in almost all studies, with variations in flavonoid content. These slight variations along with differences in total content could also be responsible for the differences in the exhibited activity.

Nevertheless, the importance of the synergistic effect of its phenolic composition as an important factor to these results also should be considered. However, there is a lack of information about interactions between phenolic compounds and its effect on the observed antioxidant activity of plant extracts. Furthermore, in studies where extracts were evaluated in food matrices, phenolic content was not characterized, hindering the analysis of the relation between bioactivity, composition, and synergism. The matrices of the study were mainly meat and related products, emphasizing the need for the consideration of a broader spectrum of food products.

### 3.3. Antimicrobial Capacity of Phenolic Compounds from Aromatic Plants

Aromatic plant’s extracts have shown great antimicrobial activity throughout the literature, as has been presented in Table 4. To study the antimicrobial capacity of plant extracts, Müeller–Hinton surface tests were mainly carried out in order to calculate the minimum inhibitory concentration (MIC50) of spice extracts, which represents the concentration inhibiting the growth of 50% of bacteria. In all the selected studies, spices’ extracts were obtained using traditional methods, infused with water, and added to different foods in order to analyze and compare their *in vitro* antimicrobial capacity.

In Gonelimali et al. (2018), the effectiveness of rosemary against Gram-positive bacteria (*Bacillus cereus, Staphylococcus aureus*), Gram-negative bacteria (*Escherichia coli, Salmonella enteritidis, Vibrio parahaemolyticus* and *Pseudomonas aeruginosa*), and fungus (*Candida albicans*) was assessed [31]. The results of diffusion in agar wells showed that plant extracts significantly affected cell membranes of Gram-positive and -negative bacteria, having great value as a natural antimicrobial. Water and ethanol as solvents were compared, showing that a rosemary ethanolic extract exhibited an inhibitory effect against four pathogenic bacteria strains (*Escherichia coli, Salmonella enteritidis, Bacillus cereus,* and *Staphylococcus aureus*) while the aqueous extract was only effective against three strains (*Escherichia coli, Bacillus cereus,* and *Staphylococcus aureus*). However, the highest MIC values (% *w*/*v*) were obtained from water-obtained extracts [31].

In Çelik et al. (2021), the antimicrobial activity of thyme extracts was evaluated through MIC values in Muller–Hillton agar using the agar-well diffusion method [56]. The assayed extract achieved inhibition of *Bacillus cereus* 702 Roma and *Mycobacterium smegmatis* ATCC607. In Bouymajane et al. (2022), antimicrobial assays were carried out using the microdilution method in tryptone soy yeast to evaluate the minimal inhibition concentration and minimal bactericidal concentration (MIC and MBC) [67]. Thus, *Thymus zygis gracillis* presented an antimicrobial effect on *Escherichia coli, Pseudomonas aeruginosa, Salmonella typhimurium, Listeria monocytogenes, Enterococcus faecalis,* and *Staphylococcus aureus.* Another thyme extract was evaluated in Mascoloti et al. (2022), where extracts were able to inhibit the growth of *Escherichia coli, Salmonella typhimurium, Enterobacter cloacae, Staphylococcus aureus, Bacillus cereus,* and *Listeria monocytogenes* [68].

Ginger extracts have proved to inhibit the proliferation of different bacteria, fungi, and viruses in the previous literature. These effects could be mainly related to the suppression of bacterial biofilm formation, ergosterol biosynthesis, and viral adhesion and internalization [69]. In Chang, Wang, Yeh, Shieh, and Chiang (2013), the antimicrobial capacity of *Zingiber officinale Roscoe* against human respiratory syncytial virus (HRSV) was studied using ELISA, where ginger was able to decrease the virus proliferation in respiratory mucosa cell lines [71]. In Zhong et al. (2021), the antimicrobial capacity of different fractions of ethanolic extracts were evaluated in comparison to essential oils [73]. Fractions were obtained by a combination of different aqueous ethanolic extracts evaporated and redissolved in water-saturated EtOAc. The EtOAc soluble fraction of ethanolic extracts presented significant antibacterial activity, while the insoluble fraction was inactive. Then, the extract was fractionated via silica gel column chromatography into eight parts according to their polarities to study them separately (fractions M1–M8). M1-M4 fractions showed potential to suppress the growth of B. subtilis while M3 presented the lower MIC value (1 μg/mL), and M2 showed higher inhibition with a MIC value of 8 μg/mL against the same bacteria. Fractions M5-M8 were inactive at a concentration of 128 µg/mL against all bacteria tested. Among the four active fractions (M1–M4), M3 showed the highest efficacy against the tested microorganisms. This high antibacterial activity could be related to its great content in bioactive components.

Clove extracts have also been studied for their antimicrobial potential. In this regard, Mostafa et al. (2018) evaluated different ethanolic plant extracts against *Bacillus cereus, Staphylococcus aureus, Escherichia coli, Pseudomonas aeruginosa,* and *Salmonella typhi* [35]. Among the extracts considered, clove ethanolic extract, along with *Punica granatum*, showed the best antimicrobial activity in fish-derived products. Additionally, their antimicrobial properties are further demonstrated in Rosarior et al. (2021), where clove extracts showed an *in vitro* antimicrobial effect against urinary tract infections (UTI) caused by *Proteus mirabilis* and *Staphylococcus epidermidis* [40]. This bioactivity has been exceptional for their aqueous extracts. At the same concentration of eugenol, the clove extract showed better antioxidant and antimicrobial capacity than its respective essential oil. This could be related to differences in phenolic content, as essential oils tend to present more apolar molecules as well as a lower concentration.

Overall, it could be considered that their antimicrobial activity depends on the bioactive compound concentration: when it is low, phenolic compounds inhibit microbial enzyme activity, whereas at high concentrations, they induce denaturation of proteins. The nature of functional groups present in these compounds should also be considered, as it varies between different phenolics. For example, hydroxyl groups could cause the decay of a proton motive force, depletion of an ATP pool, and consequently, cell death [74].

As observed, this bioactivity places the studied extracts as potential additives for food products. Moreover, these properties could also be considered for their use in food packaging. Once pathogens adhere to the food surface and form biofilms, their removal is difficult. Therefore, the development of antimicrobial packaging, using extracts, could improve food quality and safety, thus the use of these extracts has great potential in the food industry. For this reason, and in relation to new lines of research, the manufacture of antibacterial food packaging, where plant extracts are isolated or combined with photodynamic inactivation (PDI), can be profitable and sustainable, as discussed in Olszewska, Gędas, and Simões (2020) [75].

### 3.4. Structure–Bioactivity Relationship

Antioxidant activity of phenolic compounds has been related to its structure. Its observed biological activity has been associated with the compounds phenolic –OH group, hydrophobic properties associated with the benzene ring, the methyl and isopropyl groups, the binding affinity for the guanine residue of DNA, and the anti-inflammatory, apoptotic, and disruptive activities on the cell membrane [76]. Additionally, the presence of a second hydroxyl or an amino group in o- or p-position are related to a better antiradical and antioxidant performance [77]. The number and position of these hydroxyl groups and their influence on bioactivity has been established in the literature, as related to their strong donating effect and formation of a stable quinone [78,79]. In this scenario, antioxidant activity could depend on interactions between phenolic compounds and bacterial cells’ surfaces, unrelated to a specific phenolic group.

In the studied extracts, the ortho position (catechol groups) among the aromatic phenolic ring seems to be the predominant configuration for most of the phenolic acids and other phenolic compounds identified. The presence of these multiple –OH groups is prominent in compounds, such as rosmarinic acid or luteolin, rutin, naringenin, catechin, and their derivatives.

Rosmarinic acid presents in its structure two ortho-dihydroxy groups (catechol structure), which could be related to its high antioxidant activity. This compound has been found in most studied spices, showing high concentrations especially in those belonging to the *Lamiaceae* family. Oregano and rosemary also present high levels of caffeic acid and other caffeoyl derivatives. Having a highly similar structure to rosmarinic acid, their presence is related to a higher scavenging activity in the two *Lamiaceae* spices. Caffeic acid has also been found in ginger extracts [80].

Phenolic diterpenes, such as carnosol and carnosic acid, also present a catechol structure in their aromatic ring. Consequently, their presence in certain extracts can potentially impact their attributed antioxidant activity. From other spices belonging to the *Lamiaceae* family, rosemary shows the highest concentrations of these phenolic compounds.

Flavonoids are also found in the mentioned extracts. In this case, their antioxidant activity results from the location and presence of hydroxyl and oxo groups and double bonds [81,82]. Specially, some combinations have been related to higher biological activity. The presence of a 4-oxo group with either an unsaturated bond between C2 and C3 or OH groups near C3 and C5 has correlated with better bioactivity [83]. This has been observed in some molecules found in these extracts, such as apigenin, quercetin, luteolin, and rutin. Additionally, compounds, like luteolin, also present a catechol group in the B-ring, which could enhance their bioactivity [83].

The mentioned flavonoids have been identified in rosemary, thyme, oregano, and clove. However, the variation in the concentration of flavonoids and other compounds (like protocatechuic and chlorogenic acid) in different studies (which could be related to the extraction conditions and other factors previously discussed) could lead to a reduced contribution to the studied activity.

Even though the considered *Lamiaceae* extracts appear to have a similar profile, rosemary seems to exhibit higher antioxidant activity throughout different studies. This difference could be related to the higher concentration of important antioxidant molecules in its extracts, as mentioned for rosmarinic acid, or the synergistic effect of its specific polyphenolic combination as compared to oregano or thyme.

As for the antioxidant activity in clove extracts, this has been related to their composition of gallic acid, flavonols, and hydrolysable tannins. The high concentration of gallic acid specifically and its derivatives, which present three hydroxy groups in their structure, could be related to their high antioxidant activity, reported as the highest in comparison to other 26 spices [36].

### 3.5. Comparison of Spice Extracts with Other Common Additives

As these extracts seem to have an outstanding antioxidant potential, it is interesting to consider its comparative study with additives already in use, both natural and synthetic, for its evaluation for use in the food industry. Also, analyzing spices in food matrices offers important information about potential factors that could interfere in the final products.

Methanolic thyme extracts have already shown greater antioxidant activity than known additives BHA, TBHQ, and α-tocopherol [34]. As for extraction solvents, the best polyphenolic extraction from thyme has been achieved using lower concentrations of ethanol with MAE [38]. In Bouymajane et al. (2022), antioxidant capacity of extracts from aereal parts of *Thymus zygis* subsp. *gracillis* was evaluated using DPPH, establishing an IC50 of 0.234 ± 0.001 mg/mL, higher than the reference standard BHT [67].

In Jayathilakan, Sharma, Radhakrishna, and Bawa (2007), the antioxidant activity of clove (*Eugenia caryophyllata*) was evaluated in meat, both cooked and chilled, and compared with the antioxidants TBHQ and BHA [62]. Results showed that antioxidant activity presented the following order: clove (finely pulverized) > ascorbic acid > cinnamon (finely pulverized) and TBHQ > BHA. In the present study, as in the majority where the antioxidant and antimicrobial capacity of spices in food are compared, sensory characteristics, such as meat color and flavor, are also evaluated, with the aim of analyzing the acceptance of the final product with natural additives by consumers. Fresh aromatic plants are more popular, but dry extracts contribute more to the biological value [30].

Additionally, the antimicrobial capacity of clove extracts has been evaluated in comparison with the additive TBHQ and ethanol [46]. MIC values (μg/mL) for Gram-positive and -negative bacteria, respectively, were 50 and 100 for an ethanolic clove extract, 25 and 50 for TBHQ, and 50 and 100 for ethanol. A relation between a higher amount of total phenolic compounds and a relatively high antibacterial activity was observed. It was also concluded that the extraction of bioactive chemicals with high polarity solvents was more effective than extraction with low polarity solvents.

Regarding clove, eugenol is its main bioactive compound, found in concentrations ranging between 9381 mg and 14,650 mg per 100 g of fresh plant material [84]. Compared with BHT, cloves (sprouts) were the spice with the highest antioxidant activity (168.660 mmol of Trolox/100 g dry weight) and polyphenol content (14.380 gallic acid equivalents/100 g dry weight) [36]. Similar results have been observed on cooked beef patties, where clove extracts presented an increased antioxidant potential on protein and lipid oxidation when compared with BHT and ascorbic acid [63].

The antioxidant capacity of rosemary extract included in pork patties was evaluated using vitamin E as a control [60]. This was further confirmed by Sebranek et al. (2005), where the antioxidant effect of rosemary extracts on frozen raw sausages also proved to be higher than BHA and BHT [61]. Results showed that the extracts retarded lipid oxidation during meat processing. Also, in Zahid et al. (2020), ascorbic acid was used to evaluate the antioxidant capacity of cloves in cooked beef patties using the TBARS method [63]. It was also concluded that the addition of clove as a natural antioxidant could reduce the oxidation of proteins and lipids and improve the organoleptic quality of the food product.

Natural compounds used as additives in food could be a viable alternative to address the problem of microbial resistance, constitute new sources of antioxidant compounds, and hamper the negative effects of some synthetic compounds. Also, plant extracts have the added value of meeting the requirements of food safety because spices are GRAS substances and do not have a negative impact on nutritional and sensory attributes of foodstuffs, providing that the appropriate content should be taken into account for this purpose [82]. However, there is still a lack of knowledge regarding their inclusion on different food matrices and the evaluation of both their antimicrobial and antioxidant activities on these formulations. In this sense, studies should also take into consideration the implications of the introduction of phenolic extract on food color, flavor, odor, bitterness, and astringency, and its impact on consumer acceptance should be evaluated. Additionally, it is necessary to address the stability of these bioactive compounds in formulations during food storage time that could present a limitation on their applicability.

## 4. Conclusions

In the present systematic review, the literature regarding medicinal and aromatic plants’ extracts has been evaluated. An abundance of extraction techniques has been applied through the literature for the obtention of related extracts. In this sense, the development of protocols for environmentally friendly techniques is an area of study of interest for the future of the food industry. These extracts present an interesting bioactive compound content. While this has been evaluated for some extracts, a lack of individual and detailed characterization of polyphenols has been found in some studies, which may have hindered the study of the potential correlation of a specific phenolic composition with their observed antioxidant capacity.

Nevertheless, a high bioactivity associated with their phenolic content has been observed for the considered spices. While there is a clear association between phenolic compounds and antioxidant capacity, this same technological function is not attributed to a single isolated phenolic compound, but to several together, which act in synergy. On the other hand, relationships between phenolic content in food and antimicrobial capacity is not yet clear, although a higher amount of those compounds may be related to higher activity. The effect of spices on food pathogens depends on the phenolic content, but also on the interaction with other food components.

Spices’ extracts thus present great potential for their use in the food industry. When transferring the use of plant extracts as antioxidant and antimicrobial material in the food industry, we have to take into account two important aspects: (1) It has to be considered that phenolic compounds included in food could contribute to the color, flavor, odor, bitterness, and astringency, so it is important to study and control the dose of plant extracts added to food; and (2) careful considerations should be given to the stability of bioactive compounds during food storage time. In this regard, spices could be an attractive alternative not only for improving food preservation, but also to help decrease their sensory effect in certain foods.

## Figures and Tables

**Table 1:** Phenolic compounds characterized in different spices.

Spice Extract	Phenolic Compound	Extraction Technique	Extraction Conditions	Characterization Technique	References
Oregano	Glycosylated flavonoids: kaempferol-O-glucuronide and luteolin-7-O glucuronide.	MAE.	100% water, 42 °C and 2 min. extraction time.	Folin–Ciocalteu.	[38]
	Rosmarinic, caffeic, chlorogenic, ferulic, gallic, *p*-coumaric, syringic and vanillic acids.	Dynamic sonication assisted ethanol extraction.	60% ethanol (*v*/*v*) at room temperature, 0.25 h.	LC × LC-MS.	[48]
	Caffeic and caffeic-O-hexoside, protocatechic, rosmarinic, 3-, 4- and 5-O-caffeoylquinic, coumaroylquinic, ferulic-O-hexoside, ferulic, *p*-coumaric, homovanillic-O-hexoside, gallic acids, syringic, *p-* and *m*-hydroxybenzoic, kaempferol-3-O-glucoside, kaempferol, and quercetin.	SPE.	Hydroalcoholic solvent.	Folin–Ciocalteu, HPLC LTQ-Orbitrap mass spectrometry.	[43]
	Gallic acid, caffeic acid, gentisic acid, *p*-coumaric acid, vannyl acid, ferulic acid, syringic acid, catechin, rutin, quercetin, apigenin, naringenin, and eriodictyol	UAE.	60% methanol, 60% acetone, water and ethyl acetate/water [60:30, *v*/*v*].	RP-HPLC and Folin–Ciocalteu.	[24]
Rosemary	Carnosoic acid and rosmarinic acid.	SFE.	40 °C and 300 bar.	GC-FID, TLC	[32]
	Rosmarinic acid, caffeine, 7-methylrosmanol, rutin, naringin, and ferulic acid.	UAE.	Glicerol:choline chloride (1: 2 *v*/*w*) and water (10% *w*/*w*), lactic acid:choline chloride (1:3 *v*/*w*) and water (10% *w*/*w*), 1,2-propanediol:choline chloride (1:2 *v*/*w*) and water (10% *w*/*w*): deep eutectic solvents compared to pure ethanol.	Folin–-Ciocalteu.	[29]
	Phenolic acids (gallic acid, rosmarinic acid, *p*-coumaric acid), flavonoids (quercetin 3-O-galactoside, kaempferol 3-O-glucuronide), and terpenoids (rosmanol, carnosol, carnosic acid).	UAE and CE.	100% water and 50:50 ethanol:water.	Folin–Ciocalteu and Triple TOF-LC-MS-MS.	[37]
	Flavonoids (cirsimaritin, genkwanin), rosmarinic acid and terpenoids (rosmanol, carnosol, carnosic acid).	UAE and MAE.	UAE: 140 W MAE: N_2_ 20 bar, 100 °C.	HPLC-DAD-MS-TOF.	[23]
	Total polyphenols, flavonoids, anthocyanins, monomeric and condensed anthocyanins.	MAE.	Methanol: water (70:30, *v*/*v*), 70 °C.	Folin–Ciocalteu.	[26]
	Flavonoids (luteolin, apigenin, diosmetin, cirsimaritin, genkwanin) and terpenoids (rosmanol, carnosol, carnosic acid).	SFE and PLE.	SFE: 150 bar 6.6% ethanol and 400 bar with CO_2_, 40 °C. PLE: ethanol 150 °C and water 100 °C and 200 °C, 20 min.	HPLC-DAD-MS.	[45]
	Caffeic and caffeic-O-hexoside, protocatechic, rosmarinic, 3-, 4- and 5-O-caffeoylquinic, coumaroylquinic, ferulic-O-hexoside, ferulic, *p*-coumaric, homovanillic-O-hexoside, gallic acids, syringic, *p-* and *m*-hydroxybenzoic, kaempferol-3-O-glucoside, kaempferol, and quercetin.	SPE.	Hydroalcoholic solvent.	Folin–Ciocalteu. HPLC + LTQ-Orbitrap mass spectrometry.	[43]
Thyme	Derivatives of hydroxycinnamic acid: caffeic acid hexoside, rosmarinic acid and derivatives of salvianolic acid-A. Glycosylated flavonoids: luteolin-7-O-glucuronide and kaempferol-O glucuronide.	MAE.	150 °C, 50% ethanol and 9.5 min.	Folin–Ciocalteu.	[38]
	Rosmarenic acid, methyl rosmarnate, caffeic acid, cinnamic acid, chlorogenic acid, quinic acid, and flavonoids such as ferulic acid, apigenin, luteolin, and quercetin.	Maceration.	Metanol, ethanol, diethyl ether and hexane, 72 h.	Folin–Ciocalteu and HPLC.	[34]
	Caffeic and caffeic-O-hexoside, protocatechic, rosmarinic, 3-, 4- and 5-O-caffeoylquinic, coumaroylquinic, ferulic-O-hexoside, ferulic, *p*-coumaric, homovanillic-O-hexoside, gallic acids, syringic, *p-* and *m*-hydroxybenzoic, kaempferol-3-O-glucoside, kaempferol, and quercetin.	SPE.	Hydroalcoholic solvent.	Folin–Ciocalteu. HPLC + LTQ-Orbitrap mass spectrometry.	[43]
	*p*-coumarinic acid, kaempferol, epigenin, ferulic acid, luteolin, rosmarinic acid, gallic acid, epigenin, eugenol, quercetin.	UAE and CE.	Water 100% and 50:50 ethanol:water.	Folin–Ciocalteu and Triple TOF-LC-MS-MS.	[37]
Ginger	Total soluble solids, total polyphenols, flavonoids and non-flavonoids, total carotenoids, and vitamin C.	UAE.	Water as solvent, 40–70 °C and 30 min	Folin–Ciocalteu.	[22]
Pepper	Capsaicinoids (nordihydrocapsaicin, capsaicin, dihydrocapsaicin, homocapsaicin, and homodihydrocapsaicin).	UAE.	Methanol as solvent, 50 °C and 10 min.	HPLC-fluoresncence.	[44]
	Piperine and piperine acid, anisic acid, methyl anisate, shikimic acid, methyl shikimate.	Oxidative or steam distilled.	Piperine extraction: Ethanol, 80 °C, 2 h. Piperine acid extraction: Piperine dissolved in KOH/ethanol at ¼ (*v*/*v*), at 8 °C, 15 h.	^1^H NMR, ^13^C NMR, FTIR, and HPLC.	[50]
Clove	Tannins, phenolic compounds, terpenoids, glycosides. cardiac. TPC (mg/g): Kaempeforl 5.839, Catechin 0.0184, Gallic acid 0.0169.	Maceration.	80% ethanol at a ratio of 1:5 (*w*:*v*) 24 h.	GC-MS and HPLC-DAD.	[40]

DPPH: diphenyl picril hydrazyl; HPLC: high-performance liquid chromatography; MAE: microwave-assisted extraction; MS: mass spectrometry; SFE: supercritical fluid extraction; SPE: solid-phase extraction; TLC: thin-layer chromatography; TOF: time-of-flight mass spectrometry; UAE: ultrasound-assisted extraction.

**Table 2:** Antioxidant capacity of different spices related to their phenolic compounds.

References	Spice	TPC (mg GAE/g. DW)	ABTS (mmol TE/g. DW)	DPPH (mmol TE/g. DW)	FRAP (mmol Fe^2+^/mg.)	Phenolic Compounds
[36]	Oregano (*Origanum vulgare* L.)	101.7 ± 0.10	1.010 ± 0.009	-	-	Phenolic acids (caffeic acid, *p*-coumaric acid, rosmarinic acid, caffeoyl derivatives), volatile compounds (carvacrol), and other flavonoids.
	Rosemary (*Rosmarinus officinalis* L.)	50.7 ± 0.36	0.378 ± 0.00021	-	-	Phenolic acids (caffeic acid, rosmarinic acid, caffeoyl derivatives), phenolic diterpenes (carnosic acid, carnosol, epirosmanol), volatile compounds (carvacrol) and other flavonoids.
	Thyme (*Thymus vulgaris* L.)	45.2 ± 0.06	0.381 ± 0.00003	-	-	Phenolic acids (gallic acid, caffeic acid, rosmarinic acid), volatile compounds (thymol), phenolic diterpenes, flavonoids.
	Clove	143.8 ± 0.06	1.67 ± 0.00024	-	-	Phenolic acids (gallic acid), flavonol glucosides, phenolic volatile oils (eugenol, acetyl eugenol), tannins.
[30]	Rosemary	5.15 ± 0.08	-	1.30 ± 0.03	2.64 ± 0.09	caffeic acid, rosmarinic acid and flavones.
	Pepper	1.77 ± 0.02	-	1.199 ± 0.044	0.81 ± 0.62	flavones and flavones.
[41]	Rosemary	0.204 ± 0.002	8.12 ± 0.17	-	-	carnosic acid, carnosol (liquid methanol).
[43]	Oregano	-	1.34 ± 0.13	0.78 ± 0.07	-	Phenolic acids (rosmarinic, vanillinic, syringic, caffeic, protocatechic, chlorogenic, coumaric acids) and flavonoids (apigenin, hesperetin, naringenin, quercetin, among others).
	Rosemary	-	2.39 ± 0.17	1.98 ± 0.17	-	Phenolic acids (rosmarinic, vanillinic, syringic, caffeic, protocatechic, chlorogenic, coumaric acids) and flavonoids (apigenin, hesperetin, naringenin, quercetin, among others).
	Thyme	-	1.38 ± 0.13	1.15 ± 0.06	-	Phenolic acids (rosmarinic, vanillinic, syringic, caffeic, protocatechic, chlorogenic, coumaric acids) and flavonoids (apigenin, hesperetin, naringenin, quercetin, among others).
[38]	Oregano	-	0.361 ± 0.14	5.54 ± 0.52 ^b^	-	Phenolic acids (caffeic and rosmarinic acids), flavonoids (gallocatechin, galangin, lucenin-2, luteolin-O-hexoside, and luteolin-7-O-glucuronide).
	Thyme	-	3.92 ± 0.61	8.58 ± 1.75 ^b^	-	Phenolic acids (Caffeic acid hexoside, rosmarinic acid and derivatives of salvianolic acid A), flavonoids (luteolin-7-O-glucuronide and kaempferol-o-glucuronide).
[52]	Thyme (*Thymus serpyllum)*	112.27 ± 16.75	271 ± 8	-	-	Phenolic acids (rosmarinic, syringic, vanillinic, chlorogenic, *p*-coumaric, caffeic acids), flavonoids (luteolin glucoside, luteolin-glucuronide, eriodictyol-glucuronide, apigenin-glucuronide).
[51]	Thyme (*Thymus vulgaris)*	15.13 ± 0.313	-	29.22 ± 0.385 ^b^	30.88 ± 0.02 ^a^	Phenolic acids (cinnamic, rosmarinic, chlorogenic, syringic, coumaric acids), flavonoids (rutin, quercetin).
[56]	Thyme	109 ± 1.98	-	8.43 ± 0.0009 ^b^	348 ± 7.89 ^c^	Simple phenols phenols (phenolic acids and coumarins) and polyphenols (flavonoids, stilbenes, lignans, tannins).
[57]	Thyme	126.7 ± 34.3	-	42.97 ± 2.10 ^b^	50.21 ± 2.400	Phenolic acids (rosmarinic, caffeic acids), flavonoids (luteolin.7.O.neohesperidoside, apigenin-7-Neohesperidoside, apigenin-7-glucuronide).
[58]	Thyme	86.19 ± 0.36	-	218.97 ± 0.28	1.11 ± 0.00085	Flavonoids (including flavones, flavanones, isoflavones, flavonols, and anthocyanidins)

Values are the means of three independent replicates ± standard deviation (SD). ABTS: cationic radical; 2,2′-azino-bis-(3-ethyl benzothiazoline-6-ammonium sulfonate); BHA: Butylhydroxyanisole; BHT: butylhydroxytoluene; DPPH: 2,2-diphenyl-1-picrylhydrazyl free radical; FRAP: ferric iron-reducing reagent; MAE: microwave-assisted extraction; PG: propylene glycol; SFE: supercritical fluid extraction; TBARS: substances reactive to thiobarbituric acid; TBHQ: tert-butylhydroquinone; TE: Trolox equivalents; TPC: total phenol compounds; US: ultrasound-assisted extraction; WD: dry extract; WOF: release of iron on hot taste. ^a^ Data expressed in μM Fe^+2^/g. ^b^ Data expressed in EC50 μg/mL, ^c^ Data expressed in mmol TE/g.

**Table 3:** Antioxidant capacity of different spices in a food matrix, compared with other common additives.

Spice	Antioxidant Capacity Method	Food Matrix	Common Additives	Observed Effect	References
Rosemary *(Rosmarinus officinalis)*	TBARS, hexanal, vitamin E, and sensory panel.	Pork patties precooked during storage under retail conditions (10 days, 4 °C, atmospheric air).	Vitamin E	Extracts improved the retardation of lipid oxidation during meat processing	[60]
Rosemary *(Rosmarinus officinalis)*	FRAP, ABTS.	Frozen chicken nuggets.	-	Extracts improved lipid oxidative stability without altering physical or chemical characteristics during storage	[41]
Rosemary *(Rosmarinus officinalis)*	TBARS, color, and sensory panel.	Pork sausages frozen, precooked, frozen, and chilled fresh pork sausages.	BHA/BHT.	Extract was more effective than BHA/BHT for preventing a TBARS increase and red color loss in raw frozen sausage	[61]
Clove (*Eugenia caryophyllata*), cinnamon (Cinnamomum zeylanicum), and synthetic antioxidants.	WOF and non-haem iron release.	Cooked and refrigerated stored meats of three common domestic species (sheep, beef, and pork).	TBHQ, BHA, and PG.	Clove extract showed higher antioxidant activity in the studied matrices during storage than BHA and ascorbic acid	[62]
Clove (*Eugenia caryophyllata*)	TBARS.	Cooked beef patties.	BHT and ascorbic acid.	Clove extract reduced protein and lipid oxidation when compared with BHT and ascorbic acid	[63]
Water extracts of 13 spices, including oregano, cloves, and thyme	DPPH.	Processed meats.	Ascorbic acid.	Spices extracts showed greater antioxidant activity than ascorbic acid.	[47]

ABTS: cationic radical; 2,2′-azino-bis-(3-ethyl benzothiazoline-6-ammonium sulfonate); BHA: butylhydroxyanisole; BHT: butylhydroxytoluene; DPPH: 2,2-diphenyl-1-picrylhydrazyl free radical; FRAP: ferric iron-reducing reagent; MAE: microwave-assisted extraction; PG: propylene glycol; SFE: supercritical fluid extraction; TBARS: substances reactive to thiobarbituric acid; TBHQ: tert-butylhydroquinone; TPC: total phenol compounds; US: ultrasound-assisted extraction; WOF: release of iron on hot taste.

**Table 4:** Antimicrobial activity of species.

References	Spice	Extraction and/or Analytical Determination	Microorganism, Pathogen	Method for Determining Antimicrobial Activity	Food Matrix	Conclusions
[71]	Ginger (*Zingiber officinale Roscoe)*	-	HRSV.	ELISA.	-	Estimated CC50 cytotoxic concentration: 1893.8 µg/mL and >3000 µg/mL.
[31]	Rosemary *(Rosmarinus officinalis*), clavo (*Syzygium aromaticum*), and tomillo (*Thymus vulgaris)*	Conventional extraction + ultrasound. Solvents water and ethanol.	*Bacillus cereus, Staphylococcus aureus*, *Escherichia coli, Salmonella enteritidis, Vibrio parahaemolyticusy Pseudomonas aeruginosa,* and *Candida albicans*.	Diffusion in agar wells.	-	MIC (% *w*/*v*): Clove. 0.313% for BC in water. Rosemary. 1.25% for BC in water. Thyme. 2.5% for SA in water.
[46]	Clove (*Syzygium aromaticum*)	Characterization using Folin–Ciocalteu.	*Staphylococcus aureus, Listeria monocytogenes, Salmonella enteritidis, Serratia marcescens,* and *Escherichia coli.*	Mueller–Hinton surface on agar plates.	-	MIC (μg/mL): 50 for G+ and 100 for G−.
[72]	Oregano *(Origanum vulgare).*	Solid–liquid extraction and SFE.	*Staphylococcus aureus, Escherichia coli,* and *Candida albicans.*	Mueller–Hinton surface microdilution.	-	MIC (g/mL): 0.147, 0.728 and 0.311, respectively.
[50]	Pepper: Piperine and methyl piperate.	NMR, FTIR, UV-vis, fluorescence spectroscopy, and HPLC.	*S. aureus (Sa), S pyogenes (Sp) S. thypi (Sth), P. aeruginosa (Pa),* and *E. coli (Ec).*	Dissolution in DMF.	-	MIC (μg/mL) for piperine: 160 0 (*Sa*), 1200 (Sp), 400 (*Ec*), 1200 (Sth), 1600 (*Pa*). MIC (μg/mL) for methyl piperate: 2000 (*Sa*), 2400 (*Sp*), 800 (*Ec*), 2000 (*Sty*), 2400 (*Pa*).
[40]	Clove (*Syzygium aromatium*)	Characterization using HPLC.	Urinary tract infections by *Proteus mirabilis, Staphylococcus epidermidis, Staphylococcus aureus, Escherichia coli, and Klebsiella* *pneumoniae.*	Mueller–Hinton surface on agar plates. Disk diffusion assay and microwell dilution assay.	-	*Pm* (19.7 ± 0.6 mm) > *Ses* (18 mm) > *Sa* (14.7 ± 0.6 mm) > *Eci* (12.7 ± 0.6 mm) > *Kp* (12.3 ± 0.6 mm)
[22]	Ginger (*Zingiber officinalle* L.)	Extraction using UAE.	*Salmonella,* *L. monocytogenes, S. aureus.*	-	-	-
[55]	Romero *(Rosmarinus officialis* L.)	UPLC-Orbitrap-MS/MS characterization at room temperature, UV, IR, HRESI-MS, and NMR.	Gram-positive *B. subtilis.*	-	-	Analysis of the parts of the extract, against *B. subtilis*. MIC (μg/mL) for M1 16, M2 8, M3 1, M4 128, M5 > 128, M6 > 128, M7 > 128, M8 > 128
[30]	Pepper and rosemary.	Characterization using HPLC and Folin–Ciocalteu.	*Salmonella typhimurium (St), Escherichia coli (Ec), Enterococcus faecalis (Ef), Staphylococcus aureus (Sa),* and *Listeria monocytogenes (Lm).*	Mueller–Hinton surface on agar plates.	Curd.	d (inhibitory zone)/mm, respectively: Pepper: 13.3 ± 1.2 (*St*), 9.3 ± 2.3 (Ec), 15.3 ± 1.2 (*Ef*), 11.3 ± 1.2 (*Sa*), n.d (*Lm*). Rosemary: 12.7 ± 2.3 (*St*), 13.3 ± 1.2 (Ec), 19.3 ± 1.2 (*Ef*), 20.7 ± 1.2 (*Sa*), 14.0 ± 1.0 (*Lm*).
[35]	Clove, thyme, ginger	-	*Bacillus cereus (Bc), Staphylococcus aureus (Sa), Escherichia coli (Ec), Pseudomonas aeruginosa (Pa),* and *Salmonella typhi.(St).*	Mueller–Hinton surface on agar plates.	Fish.	d(inhibitory zone)/mm, Clove 14.6 ± 0.37 *(Bc),* Thyme 17.6 ± 0.31 *(Sa),* clove 11.9 ± 0.34 *(Ec),* clove 13.4 ± 0.11 *(Pa).*

*Bc: Bacillus cereus*; CC50: cytotoxic concentration causing 50% cell death; *Cp: Clostridium perfringens*; *Ec: Escherichia coli*; *Ef: Enterococcus faecalis*; ELISA: enzyme-linked immunosorbent assay; G−: Gram-negative bacteria; G+: Gram-positive bacteria; HPLC: high-performance liquid chromatography; HRSV: human respiratory syncytial virus; *Lm: Listeria monocytogenes*; MIC: minimum inhibitory concentration; *Sa: Staphylococcus aureus*; SFE: supercritical fluid extraction; *St: Salmonella typhi*; UAE: ultrasound-assisted extraction.

## Data Availability

No new data were created or analyzed in this study. Data sharing is not applicable to this article.
